# Investigating A Multi-Domain Polyketide Synthase in *Amphidinium carterae*

**DOI:** 10.3390/md21080425

**Published:** 2023-07-27

**Authors:** Saddef Haq, Benjamin L. Oyler, Ernest Williams, Mohd M. Khan, David R. Goodlett, Tsvetan Bachvaroff, Allen R. Place

**Affiliations:** 1 Institute for Marine and Environmental Technologies, University of Maryland Center for Environmental Science, 701 East Pratt St., Baltimore, MD 21202, USA; saddefhaq@gmail.com (S.H.); williamse@umces.edu (E.W.); bachvarofft@umces.edu (T.B.); 2 University of Maryland School of Medicine, 655 W. Baltimore Street, Baltimore, MD 21201, USA; benjamin.oyler@nih.gov (B.L.O.); mohsin.khan@umaryland.edu (M.M.K.); 3 Department of Biochemistry and Microbiology, University of Victoria, Victoria, BC V8S 1P7, Canada; goodlett@uvic.ca

**Keywords:** dinoflagellate, PKS, cerulenin, toxin

## Abstract

Dinoflagellates are unicellular organisms that are implicated in harmful algal blooms (HABs) caused by potent toxins that are produced through polyketide synthase (PKS) pathways. However, the exact mechanisms of toxin synthesis are unknown due to a lack of genomic segregation of fat, toxins, and other PKS-based pathways. To better understand the underlying mechanisms, the actions and expression of the PKS proteins were investigated using the toxic dinoflagellate *Amphidinium carterae* as a model. Cerulenin, a known ketosynthase inhibitor, was shown to reduce acetate incorporation into all fat classes with the toxins amphidinol and sulpho-amphidinol. The mass spectrometry analysis of cerulenin-reacted synthetic peptides derived from ketosynthase domains of *A. carterae* multimodular PKS transcripts demonstrated a strong covalent bond that could be localized using collision-induced dissociation. One multi-modular PKS sequence present in all dinoflagellates surveyed to date was found to lack an AT domain in toxin-producing species, indicating *trans*-acting domains, and was shown by Western blotting to be post-transcriptionally processed. These results demonstrate how toxin synthesis in dinoflagellates can be differentiated from fat synthesis despite common underlying pathway.

## 1. Introduction

Dinoflagellates are unicellular protists that are often implicated in harmful algal blooms (HAB), resulting in the deaths of fish and human illnesses worldwide. This is due to potent toxin production that is consistent with the polyketide origin of biosynthesis [1,2,3]. Polyketide synthases (PKS) are similar to fatty acid synthases (FAS), where individual domains sequentially add activated acetate-derived 2-C units to a growing carbon chain [4,5]. The PKS process begins when an acyl monomer is loaded onto the phosphopantetheine “arm” of the acyl carrier protein (ACP) by acyl transferase (AT) domains. The ACP is the site of monomer attachment as well as the site of condensation and reduction reactions which occur during PKS biosynthesis. Condensation reactions are carried out by ketosynthase (KS) domains, while reduction reactions are mediated by ketoreductase (KR), dehydratase (DH), and enoylreductase (ER) domains. At the end of product synthesis, the thioesterase (TE) domain is responsible for the release of the finished product [6,7,8,9].

**Figure 1:**
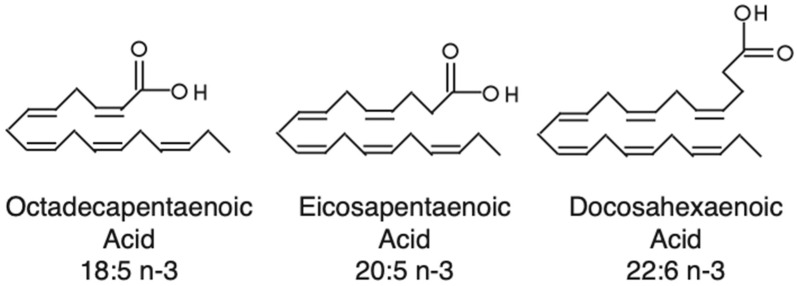
Structures of three polyenoic fatty acids synthesized by dinoflagellates.

Dinoflagellates are known to make polyenoic fatty acids like octadecapentaenoic acid (18:5n-3, i.e., 18 carbons with 5 unsaturated for every 3 carbons) [10], which were first identified from dinoflagellates (dinophytes) and, thereafter, found in raphidophytes and haptophytes (Figure 1). In these microalgae, 18:5 was shown to be confined to galactolipids [11,12]. As suggested by Bell et al. (1997) [10], 18:5n-3 might be more widespread in microalgae than previously thought, as 18:5n-3 might have been misidentified as 20:1n-9 in many studies. The most reasonable hypothesis about 18:5n-3 synthesis is the existence of a yet uncharacterized delta-3-desaturase or that it is synthesized directly as a polyunsaturated fatty acid (PUFA) via PKSs.

In addition to PKSs, non-ribosomal peptide synthetases (NRPS) and NRPS/PKS hybrids are also present in dinoflagellates [13]. NRPSs have a mechanism that is similar to PKSs; however, amino acid residues are incorporated into the product. Adenylation (A) domains recognize specific amino acids for incorporation. The thiolation (T) domains form an aminoacyl thioester intermediate, while the condensation (C) domains form the peptide bond between substrates. Finally, the thioesterase (TE) domain releases the final product [14]. PKS/NRPS hybrids combine with domains from both of these pathways [5,7]. 

**Figure 2:**
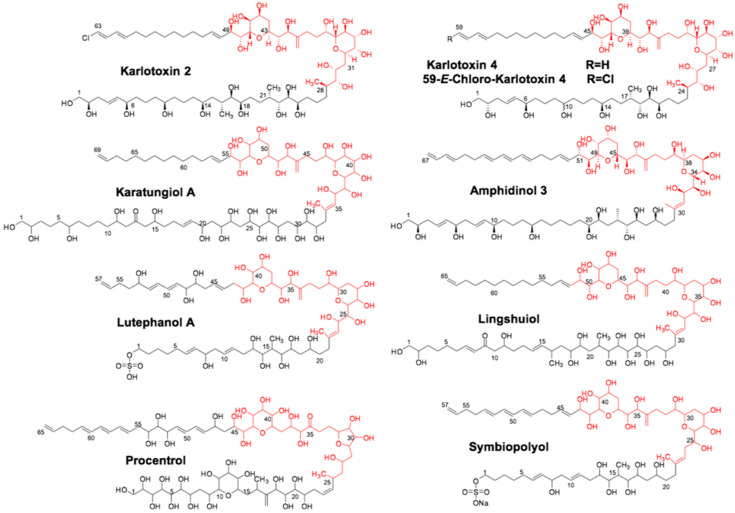
Suite of sterolysin compounds synthesized by different dinoflagellate species. Structurally similar sterolysin compounds synthesized by different dinoflagellate species in the genera *Symbiodinium, Karlodinium, Amphidinium*, and *Prorocentrum*.

Much of the current research into dinoflagellate PKSs has focused on the chemistry of the toxins themselves and their mechanism of action [5]. With an ever-increasing number of dinoflagellate transcriptome datasets becoming available, the understanding of dinoflagellate PKS/NRPS genetics and how they are different from bacterial and fungal models is rapidly growing [6,9,15,16,17]. Although the vast majority of described PKS genes occur as single domains in dinoflagellates, the goal of this study was to focus on multi-domain PKS genes since their domain arrangement gives some clues about the products they synthesize. The methods employed consisted of genetic, biochemical, and mass spectrometric tools to maximize the utility of transcriptomic datasets focusing on the basal dinoflagellate *Amphidinium carterae* (Hulbert 1957). This species is often used as a “model” dinoflagellate because it contains a smaller genome than other species in the group and is the earliest branching toxic species [18]. *Amphidinium carterae* produces amphidinols [5,19], which are members of a suite of compounds termed sterolysins (Figure 2). These pore-forming toxins create a complex with specific sterols in the cell membrane resulting in cell lysis [6,19,20]. *A. carterae* also produces other cytotoxic amphidinolides and macrolide compounds of polyketide origin that have anti-fungal and anti-cancer properties [21]. These are all presumed to be synthesized by PKS machinery due to repeated acetate units that are the hallmark of ketosynthase-based synthesis [5].

**Figure 3:**
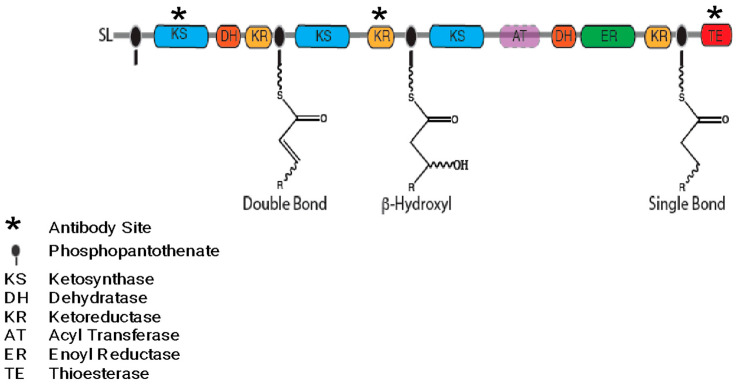
The “Triple KS” gene is shown as a multi-modular polyketide synthase (PKS) in *Amphidinium carterae*. Resultant products based on a purely processive synthesis are shown on the phosphopantotheinate site (black oval) for each module. Although the AT domain is not present in *A. carterae*, the site of the AT domain in *Akashiwo sanguinea* is depicted in pink. A * denotes domains for which complementary polyclonal antibodies were synthesized.

One multi-modular PKS termed the “Triple KS” for its three apparent ketosynthase modules and first described by Williams et al., is of particular interest in that it is ubiquitously present in dinoflagellate transcriptomes, indicating a core function [13,22]. Although the genes for classic lipid synthesis have been described elsewhere [23], other common processes that could be mediated by PKSs include PUFA synthesis and the “hairpin” structure that is found in many toxins across a wide range of Dinoflagellate species (Figure 3) [5,13]. The expression patterns of the Triple KS protein and its ability to synthesize PUFAs and/or toxins were assessed, demonstrating a complex biology with post-translational processes and a unique gene arrangement in “toxic” species. The results are discussed within the current understanding of dinoflagellate toxin synthesis and the possible ways in which the Triple KS could play a role.

## 2. Results

### 2.1. Multi-Modular Polyketide Synthase in Amphidinium carterae

The domain arrangement of a multi-modular polyketide synthase includes three consecutive KS domains, each followed by different modification domains. The estimated molecular weight of this protein is 478 kDa. The sequence ends with a thioesterase domain (TE) in *Amphidinium carterae*. Sequences similar to this were found in all dinoflagellates surveyed via transcriptome analysis [24]. However, in *Amphidinium carterae*, the acyl transferase (AT) domain was not found in a *cis* configuration with the transcript as it was in *Akashiwo sanguinea*: a dinoflagellate that does not produce a known toxin. The reactions performed by each ketosynthase (KS) module are shown on the phosphopantetheinyl-binding site (Figure 3). Antibodies used in subsequent experiments were synthesized with complementarity to the regions depicted with an asterisk (Figure 3). 

### 2.2. Thin Layer Chromatography Showing ^14^C Acetate Incorporation into Lipids in A. Carterae with and without Cerulenin Inhibition

**Figure 4:**
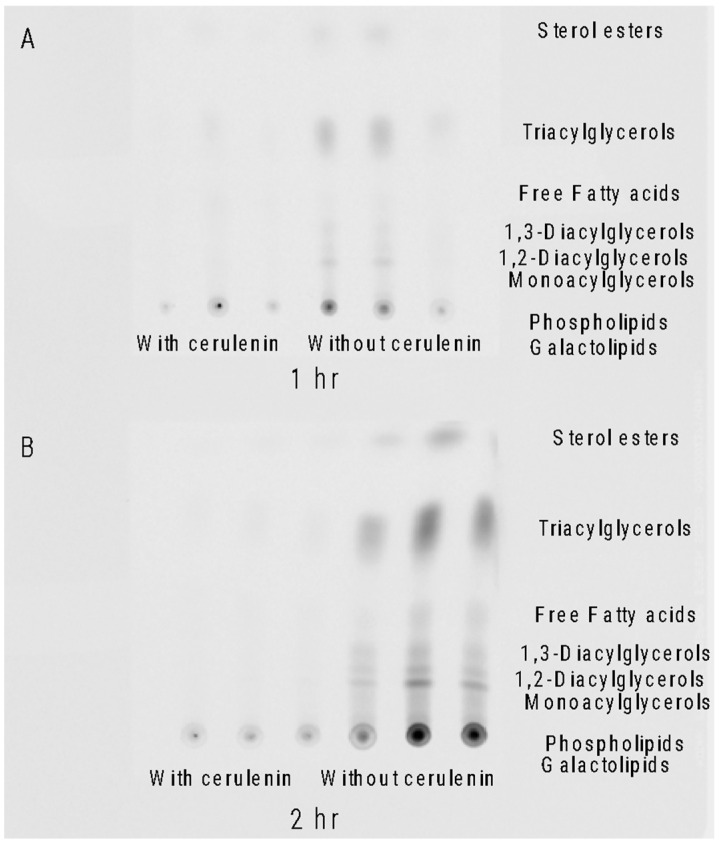
Thin layer chromatography (TLC) of extracted lipids. Triplicate lipid samples showing ^14^C acetate incorporation patterns with and without 100 μM cerulenin in *Amphidinium carterae*. (**A**) Lipid profile of *A. carterae* 1 h after ^14^C acetate addition with and without cerulenin incubation. (**B**) Lipid profile of *A. carterae* 2 h after ^14^C acetate addition with and without cerulenin incubation. Lipid classes corresponding to migration on the TLC plate are listed on the right of the image.

Cerulenin, a ketosynthase inhibitor and known disruptor of lipid synthesis, was added to *A. carterae* cultures to block KS activity. The phosphorimage of a thin layer chromatography (TLC) plate of *A. carterae* lipids showed that ^14^C acetate was not detectable in cerulenin-treated samples. At 1 h, there was incorporation into uninhibited control samples and very little incorporation into samples treated with 100 μM cerulenin (Figure 4A). After two hours there was extensive ^14^C acetate incorporation into all the lipid classes in the control samples, while the samples treated with 100 μM cerulenin showed almost no ^14^C acetate incorporation (Figure 4B). 

### 2.3. Quantitation of ^14^C Acetate Incorporation into Lipids and Amphidinols

**Figure 5:**
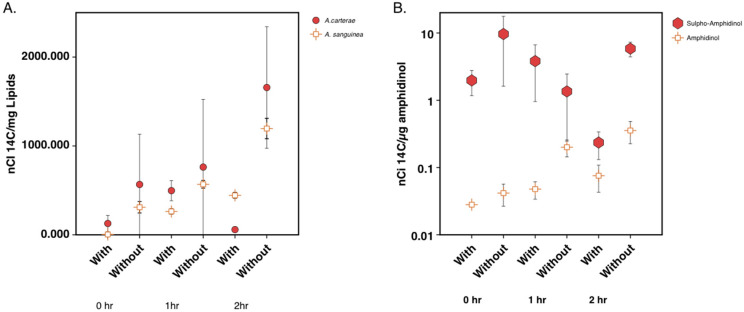
Quantitation of ^14^C acetate incorporation into lipids and amphidinol over a 2-hour period after cerulenin addition. (**A**) Quantitation of incorporation of ^14^C acetate into lipids in *A. carterae* and *A. sanguinea* at 0-, 1-, and 2 h time points with and without cerulenin addition. (**B**) Quantitation of incorporation of ^14^C acetate into amphidinol and sulpho-amphidinol at 0-, 1-, and 2-h time points with and without cerulenin addition. Error bars show one standard deviation for triplicate samples.

The ^14^C acetate incorporation into the lipids, including amphidinol and sulpho-amphidinol, was reduced over time when cerulenin was added. Lipids were extracted, and the nanocuries (nCi) of ^14^C acetate incorporation per mg lipid was calculated for *A. carterae* and *A. sanguinea*. At the 2-hour time point, *A. sanguinea* showed the ^14^C acetate incorporation of 1600 nCi/mg of the lipid in the control samples, while cerulenin-treated samples measured 450 nCi/mg of the lipid. In *A. carterae*, incorporation into the control samples was 1200 nCi/mg lipid, while cerulenin-treated samples were 60 nCi/mg of the lipid. Control samples showed increased ^14^C acetate incorporation per mg of the lipid over the 2-hour time period in both species. Cerulenin-treated samples showed a decrease in ^14^C acetate with a sharper drop in its incorporation observed in *A. carterae* than in *A. sanguinea* at 2 hours post-treatment (Figure 5A). Aqueous fractions from the lipid extraction were used to quantify toxin levels and label incorporation. In *A. carterae*, nCi of ^14^C acetate per μg amphidinol was calculated. At the 2-hour time point, control samples resulted in the ^14^C acetate incorporation of 0.35 nCi/μg of amphidinol and 5.8 nCi/μg sulpho-amphidinol. Cerulenin-treated samples at the 2-hour time point resulted in the incorporation of values of 0.07 nCi/μg amphidinol and 0.235 nCi/μg of sulpho-amphidinol. Control samples showed an increase in ^14^C acetate incorporation over time, with more incorporation into sulpho-amphidinol than amphidinol. Samples-treated with 100 μM cerulenin showed a reduction in ^14^C acetate incorporation by the 1- and 2-hour time points, with more incorporation observed into sulpho-amphidinol (Figure 5B). The percentage of ^14^C label incorporation was calculated for the total lipid and toxin fractions **(**Table 1). In all cases, a lower percent of ^14^C incorporation was observed in cerulenin-treated samples when compared to the control samples.

### 2.4. UV Absorbance Chromatograms Showing ^14^C Acetate Incorporation into Amphidinol and Sulpho-amphidinol

**Figure 6:**
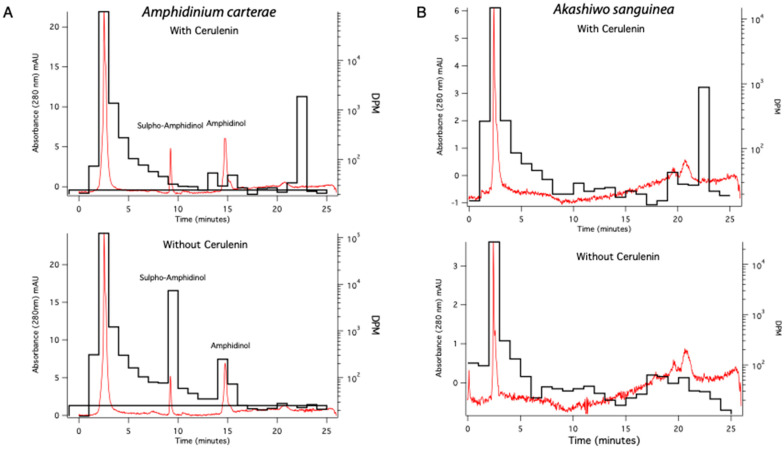
HPLC UV absorbance at 280 nm displaying amphidinol and sulpho-amphidinol (red trace) overlaid with ^14^C acetate incorporation dpm (black trace). (**A**) The red traces show the 280nm UV absorbance chromatograms for *A. carterae* extracts with amphidinol and sulpho-amphidinol peaks labeled. The black trace shows disintegrations per minute (dpm) for ^14^C acetate label incorporation into amphidinol and sulpho-amphidinol fractions without cerulenin addition. (**B**) The red trace shows the UV absorbance chromatograms at 280 nm in *A. sanguinea*. The black trace shows the dpm of ^14^C acetate in this fraction. Data shown are from 2 h sampling.

A UV absorbance chromatogram of *A. carterae* extracts, 2 h post-treatment, monitored at 280 nm, was overlaid with disintegrations per minute (dpm) to show ^14^C incorporation into sulpho-amphidinol and amphidinol (Figure 6A). Each trace began with a large peak describing the void volume, likely unincorporated acetate or polar metabolites eluting with/composed of acetate, as indicated by the high number of disintegrations per minute measured in that peak (Figure 6). The sulpho-amphidinol was eluted at 9 min, and amphidinol was eluted at 15 min. In the control samples, there was ^14^C acetate incorporation into both forms of amphidinol, with more incorporation observed in the sulpho-amphidinol form. The sample treated with 100 μM cerulenin showed no incorporation of ^14^C acetate into the toxin but showed the UV absorbance peaks for existing sulpho-amphidinol and amphidinol at 9 and 15 min, respectively. Aqueous fraction samples for *A. sanguinea* were also run as a control. *Akashiwo sanguinea* does not make amphidinols; therefore, there were no peaks in the UV absorbance chromatograms at 280 nm or with ^14^C acetate incorporation observed at the positions corresponding to amphidinol or sulfo-amphidinol (Figure 6B).

### 2.5. Quantitation of Total Toxin and Lipid Levels in A. carterae and A. sanguinea

**Figure 7:**
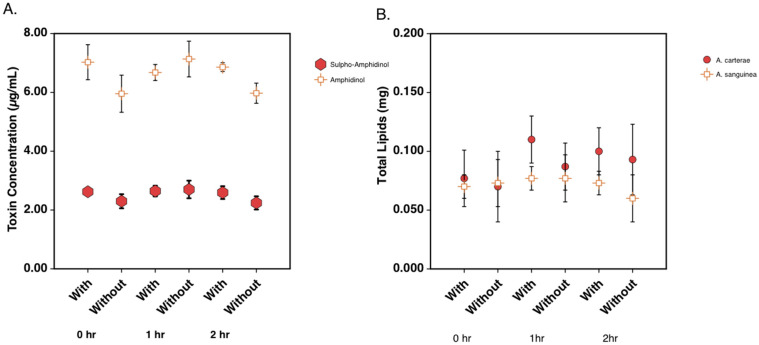
Total toxin and lipid levels in *A. carterae* and *A. sanguinea* over a 2-hour period. (**A**) Quantitation of amphidinol and sulpho-amphidinol in *A. carterae* during ^14^C acetate labeling study. (**B**) Quantitation of dry weight of extracted lipids in *A. carterae* and *A. sanguinea* during ^14^C acetate labeling study.

Toxin and lipid levels were quantified during the 2 h cerulenin labeling experiments. Relative sulpho-amphidinol and amphidinol levels were quantified using the area under the curve from LC analysis. The analysis revealed that toxin levels remained relatively constant across the time course of the experiment. The total amphidinol levels remained around 2.5 μg/mL of the culture, while sulpho-amphidinol levels remained at between 5 and 6 μg/mL of the culture (Figure 7A). Lipids were quantified using the dry weight (mg) of the extracted lipids in *A. carterae* and *A. sanguinea*. The lipid levels in both species were similar and remained between 0.07 and 0.1 mg lipids across the 2 h experiment (Figure 7B).

### 2.6. Multi-Modular PKS Transcript Abundance over a Diel Cycle in A. carterae

**Figure 8:**
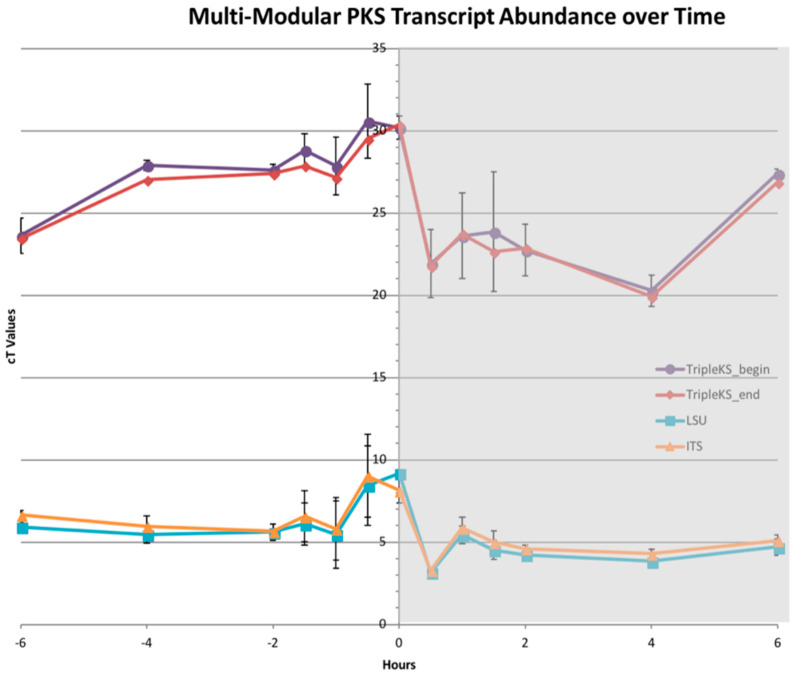
Multi-modular PKS transcript abundance across a diel cycle. Plot of C_t_ values from a RT-PCR across a diel cycle 6 h before and after lights were turned off at hour 0 (shaded region denotes when lights are off). The plot shows two primer pairs designed against multi-modular PKS sequence (purple and red) as well as 2 control proteins LSU and ITS (cyan and orange). PKS transcript levels lower than LSU and ITS, as depicted by the higher C_t_ values. All transcripts followed the same general decrease before and increase after the lights were turned off.

The transcript abundance of the multi-modular PKS showed the same pattern near the light:dark transition, which is in agreement with prior results [25,26,27]. A decrease in transcripts was observed one hour prior to when lights were turned off, and a sharp incline was observed one hour after. Transcript levels further out from this time point remained relatively steady. The two ribosomal proteins, including the internal transcribed spacer (ITS) and ribosomal large subunit (LSU), were used as controls. The transcript levels of the multi-modular PKS were much lower in comparison to the ribosomal proteins, with higher C_t_ values between 20 and 30, while LSU and ITS had C_t_ values between 3 and 9, which revealed that these were highly abundant transcripts (Figure 8). 

### 2.7. Western Blotting of Three Protein Domains in the Multi-modular PKS from A. carterae

**Figure 9:**
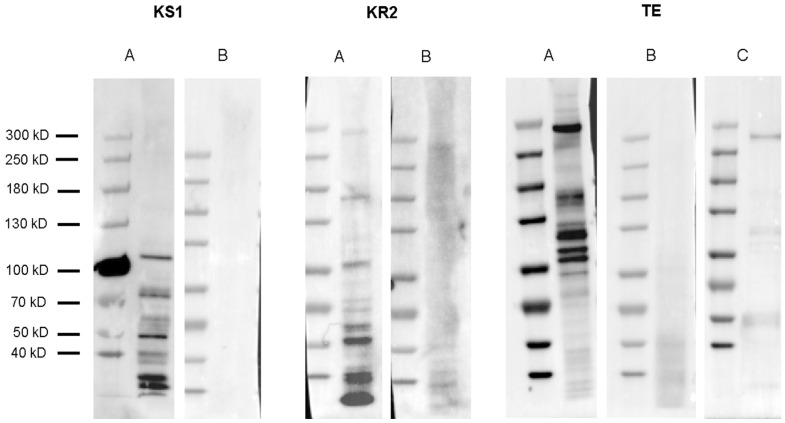
Western blotting analysis of proteins in a multi-domain polyketide synthase (PKS) in *Amphidinium carterae*. Panes A and B for each antibody refer to Western blots without and with antibody blocking epitope peptides, respectively. KS1: Proteins with the highest band at 106 kDa observed on a 300,000-cell equivalent pellet using an anti-KS1 antibody. KR2: PKS proteins with the highest band at 295 kDa observed on a 250,000-cell equivalent pellet using an anti-KR2 antibody. TE: Proteins with the highest band at 290 kDa observed on a 300,000-cell equivalent pellet using an anti-TE antibody. TE pane C: Proteins with the highest band observed at 290 kDa in a 95,000-cell equivalent native protein lysate using an anti-TE antibody.

Western blotting analysis was performed using polyclonal antibodies designed against the KS1, KR2, and TE domains of the multi-modular PKS protein (Table 2). None of the antibodies resulted in a band close to the calculated size of the full protein, 478 kDa. The highest bands observed in a cell pellet for anti-KS1, anti-KR2, and anti-TE were 106 kDa, 295 kDa, and 290 kDa, respectively. Additional bands below the highest reported band were observed for all three antibodies (“A” panes). The polyclonal antibodies were blocked with the epitope peptide to show the specificity of the antibody. All antibodies showed a reduction or complete absence of bands, demonstrating the specificity of the antibodies (“B” panes). Western blotting on a native protein lysate using the anti-TE antibody revealed the same band at 290 kDa as well as some additional bands at lower molecular weights; however, not as many were observed from the cell pellet (Figure 9C). 

### 2.8. Mass Spectra of Adducts to the Synthetic KS Peptide

**Figure 10:**
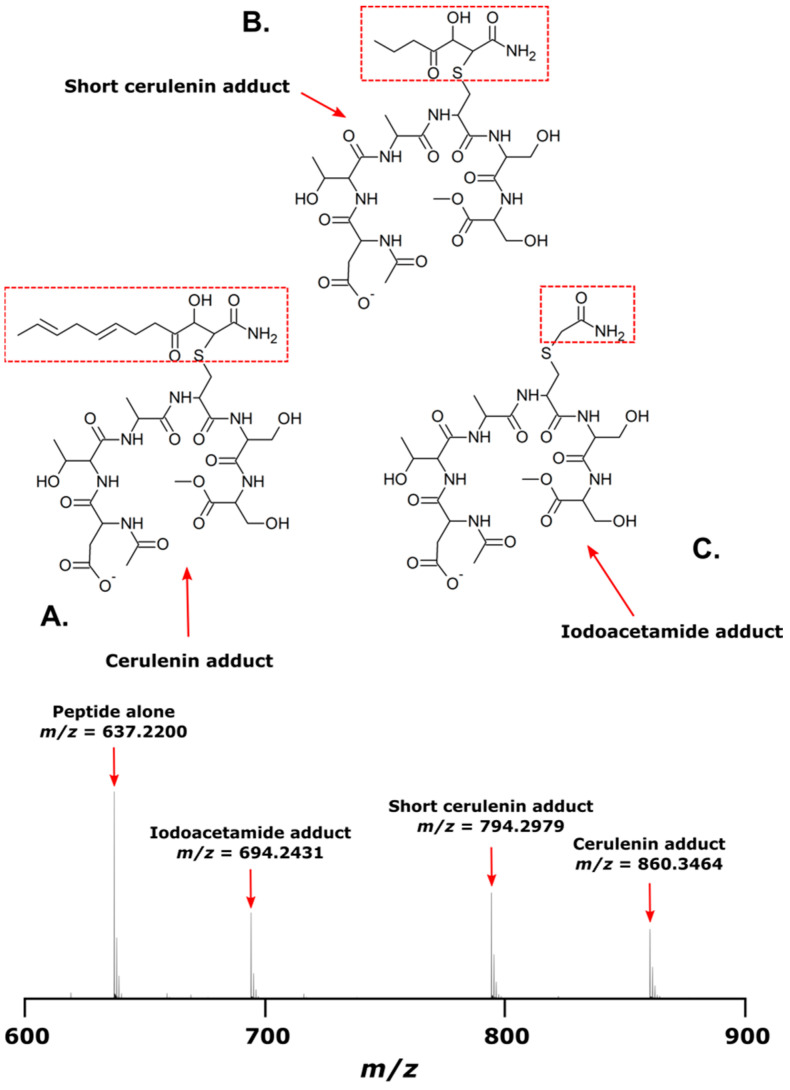
Mass spectrum of synthetic peptide, DTACSS, alone and coupled with cerulenin, a cerulenin analog, and iodoacetamide in vitro. (**A**) Structure of peptide when reacted with cerulenin; a corresponding ion in mass spectrum is seen at *m*/*z* 860.3463. (**B**) Structure of peptide when reacted with a cerulenin analog with a shorter side chain; a corresponding ion in mass spectrum is seen at *m*/*z* 794.2979. (**C**) Structure of peptide when reacted with iodoacetamide; a corresponding ion in mass spectrum is seen at *m*/*z* 694.2431. The ion detected for the peptide alone is observed at *m*/*z* 637.2200. Red boxes show the structure of adduct formation.

**Figure 11:**
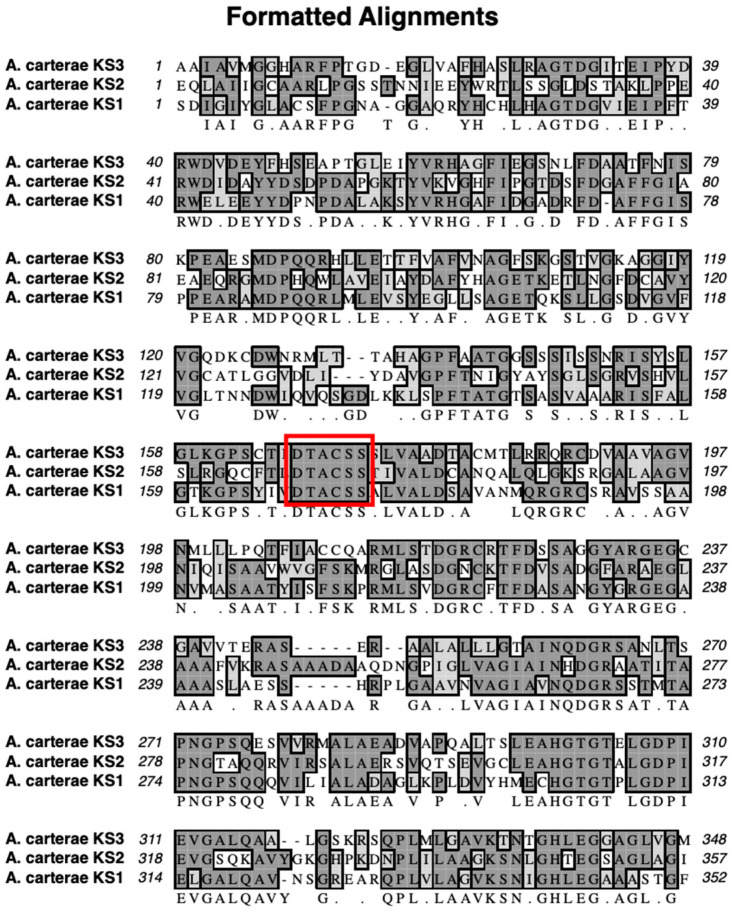
Sequence alignment of the three KS domains in the multi-modular PKS in *A. carterae*. The red box denotes the sequence that was used to synthesize the synthetic peptide, which is the conserved region that contains the active site cysteine.

**Figure 12:**
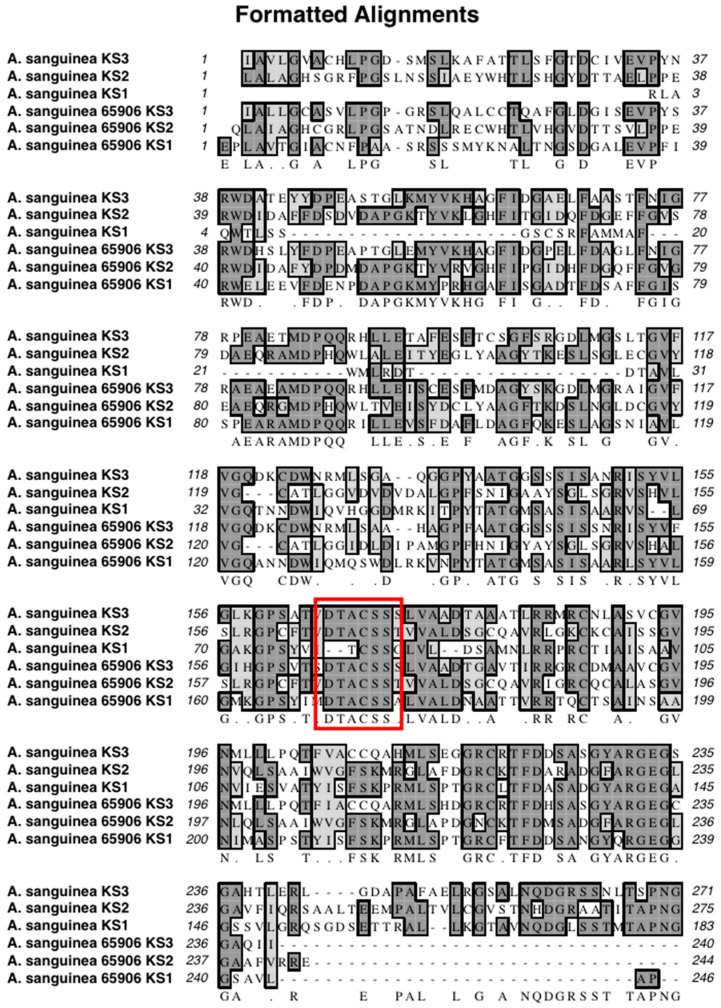
Sequence alignment of the three KS domains in multi-modular PKS in *A. sanguinea*. The red box denotes the conserved residues around the active site cysteine in these proteins.

The mass spectra of the synthetic peptide (DTACSS) derived from the conserved active site of the KS domains of the Triple KS are shown in Figure 10. Red boxes denote the inferred adduct structure with the peptide following its reaction with iodoacetamide, cerulenin, and the truncated analog of cerulenin with a shortened side chain. The sequence alignment used to generate the synthetic peptide is shown in Figure 11, with a comparison alignment to the corresponding KS domains from the *Akashiwo sanguinea* Triple KS, as shown in Figure 12. 

### 2.9. Fragmentation of the Synthetic KS Peptide and Cerulenin Adducts 

**Figure 13:**
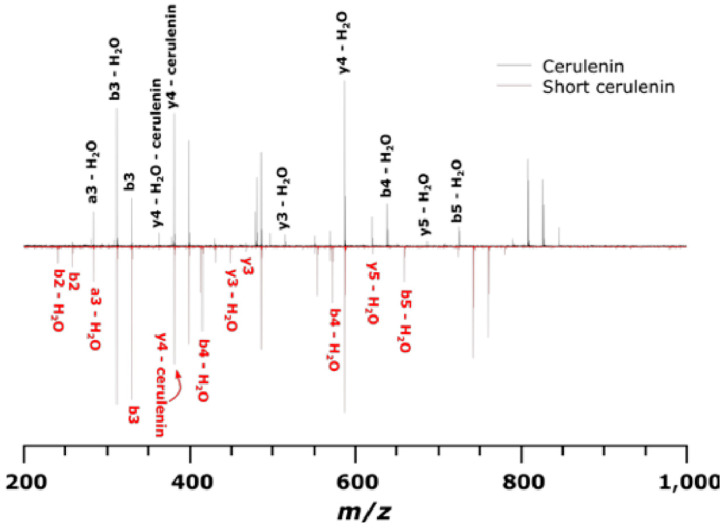
Tandem mass spectra (CID) of KS peptide reacted with cerulenin and the shorter cerulenin analog. The black spectrum denotes product ions for cerulenin-peptide while the red spectrum denotes product ions for short cerulenin-peptide. An ion followed by – cerulenin denotes an ion that has lost its respective adduct during CID.

**Figure 14:**
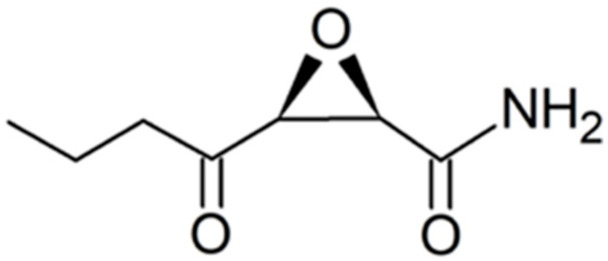
Structure of a cerulenin analog that has a shorter side chain than cerulenin.

Tandem mass spectra showing the peptide product ions from the cerulenin and short cerulenin reacted peptide is depicted in Figure 13, with the structure of the cerulenin analog shown in Figure 14. The spectra showed multiple water losses as well as b- and y-ions covalently bound to cerulenin or the cerulenin analog and indicating the location of binding at the cysteine residue. Peptide product ions with a loss of cerulenin during collision-induced dissociation (CID) are denoted as “-cerulenin” in the figure. The spectra also showed ions corresponding to the full cerulenin-reacted peptide, followed by ions corresponding to water losses in the 800–900 *m*/*z* range. 

## 3. Discussion

Toxins synthesized by polyketide synthases (PKS) in dinoflagellates are often implicated in harmful algal bloom (HAB) events; however, the pathways by which these toxins are synthesized still have not been elucidated [5,28]. With an increasing number of studies describing multi-domain PKS sequences obtained through transcriptome analysis, we focused on a triple-module PKS. This sequence was pulled from other multi-domain PKS, PKS/NRPS, and NRPS sequences because of a crucial correlation with toxicity [29]. The acyl-transferase (AT) domain was found in non-toxin-producing species but was missing in species that make toxins in a transcriptome survey on many dinoflagellate species (Figure 3) [22,24]. This feature of AT domains not being included in type I PKSs is something that has been observed in other algae. Often the AT acts in *trans* configuration to mediate the necessary acyl transferase reaction in a non-iterative fashion with the *trans*-AT type PKS [12]. 

Initially, we looked at the overall effect of ketosynthase (KS) inhibition on metabolite synthesis by using a KS inhibitor, cerulenin, and a ^14^C acetate label [30]. In the dinoflagellate *Symbiodinium*, cerulenin inhibition resulted in the significant inhibition of free fatty acids as well as phosphatidylethanolamines [31]. These experiments were then conducted on *Amphidinium carterae*, which made the toxin amphidinol, and *Akashiwo sanguinea*, which did not make an amphidinol-like compound but otherwise had the same sterol composition as *A. carterae* [32,33,34]. Labeling studies have shown that amphidinols are synthesized using glycolate as the starter molecule, and acetate provides the 2-C units for the rest of the molecule [1]. We measured the effects of cerulenin on lipids and toxins by quantifying ^14^C acetate incorporation into these compounds in the presence of cerulenin. We began by studying the effects on lipids which were expected to be inhibited with the cerulenin treatment. The phosphor imaging of a TLC plate revealed that by two hours, there was almost the complete cessation of ^14^C acetate incorporation into all classes of lipids in *A. carterae*. The control samples that did not receive cerulenin treatment showed an increase in ^14^C acetate incorporation over time (Figure 4). The quantitation of ^14^C into lipids showed the nCi of the radiation detected from ^14^C acetate per mg of lipid decreased for *A. carterae* and *A. sanguinea* lipid extracts over time, while the control samples showed an increase in their incorporation by 1 and 2-hour marks (Figure 5A). 

When we analyzed the toxins, there was a reduction in the nCi of ^14^C acetate per μg of both sulpho-amphidinol and amphidinol over time while the control samples continued to incorporate ^14^C acetate into both forms of amphidinol (Figure 5B). Interestingly, when counting for the dpm in HPLC fractions, more ^14^C acetate was incorporated into the sulpho-amphidinol than amphidinol. This suggested to us that the toxin was synthesized in the sulpho form and then later processed to amphidinol (Figure 6). The quantity of the total lipid, sulpho-amphidinol, and amphidinol levels during the experiment remained steady, suggesting that cerulenin did not decrease in its overall levels during this two-hour experiment (Figure 7). These experiments show that cerulenin is not only able to inhibit KS domains involved in lipid synthesis but also the KS domains involved in toxin synthesis. 

The third module of the multi-domain PKS (Figure 3) has the same domain arrangement as a traditional fatty acid synthase (FAS), namely KS-DH-ER-KR-TE, with only the AT domain missing in *A. carterae* [13]. However, this arrangement is different from the arrangements observed in marine γ-proteobacteria PUFA synthases that also make some of the same PUFA compounds as dinoflagellates [35]. This leaves the possibility that dinoflagellates are able to synthesize both of these products using the same machinery, and AT domains could be acting in *trans* configuration with the machinery in a non-iterative fashion. *Trans*-ATs are able to synthesize diverse bioactive polyketide products and, while still an emerging field, have been studied more thoroughly in bacterial and fungal systems to date [36,37,38]. Previous works that have identified *trans* ATs and multi-modular PKSs in dinoflagellates have hypothesized that multiple PKSs work together, and the *trans*-AT configuration could be a way to increase the diversity in the metabolites being synthesized [29]. Kohli et al. showed how fatty acid synthase genes in dinoflagellates have been observed to be more conserved, while PKSs tend to be more variable [23,36,38]. This fits well with our observation of a *cis* AT in *A. sanguinea*, while *trans* ATs are observed in *A. carterae*. The AT domain could be mediating product specificity in this scaffold, allowing for the production of both fatty acids and toxins.

The transcript abundance of the multi-modular PKS over a diel cycle reveals high C_t_ values indicating that transcripts are not highly abundant, which could suggest low protein abundance (Figure 8). Around the light:dark transition, the decrease prior to the lights being turned off, followed by a sharp increase one hour into the dark phase, provided an established pattern that has been observed in all mRNA transcripts studied to date [24,25,27]. 

To further characterize the multi-domain PKS in *A. carterae*, we synthesized antibodies to the KS1, KR2, and TE domains of this PKS sequence, hypothesizing that we would see a protein at or near the calculated size of 478 kDa. None of the antibodies resulted in a band that was consistent with the full-sized protein; however, anti-KR2 and anti-TE antibodies showed similar size bands around 290 kDa, while anti-KS1 showed a much smaller band at 106 kDa (Figure 9). For the incubation of antibodies with the peptides, they were synthesized against and resulted in a reduction or elimination of bands in cell lysates showing the specificity of the antibody to the target epitopes (“B” panes Figure 9). Multiple bands were observed in the cell lysate samples with all three antibodies, as well as in a native protein lysate when blotted with the anti-TE antibody, which we believe is a controlled degradation process that occurs in these cells (Figure 9C). The epitope sequences for all the antibodies were run against the *A. carterae* transcriptome, and the sequences were matched only to the protein of interest (Data not shown). 

To test whether cerulenin would be able to bind to the active site cysteine in the 3 KS domains in the multi-modular PKS, a peptide was synthesized with the sequence DTACSS which is the conserved KS active site in *A. carterae* (Figure 11) and *A. sanguinea* (Figure 12). When reacted with cerulenin or a cerulenin analog with a shorter side chain (Figure 14), adducts formed through their reaction with the sulfhydryl group on the cysteine residue (Figure 10A,B). A reaction with iodoacetamide, as an alkylating agent, was conducted as a control and resulted in an adduct on the same sulfhydryl group (Figure 10C). The collision-induced dissociation (CID) of the peptide showed that the adduct was covalently bound to the cysteine, and most product ions still had cerulenin or the cerulenin analog attached (Figure 13). Given the strong bond of the cerulenin with this KS active site peptide, we postulate that these three KSs in the multi-domain PKS were all inhibited in our initial acetate labeling experiments, consequently inhibiting the ^14^C acetate incorporation into lipids in *A. sanguinea* and into lipids and amphidinols in *A. carterae*.

In dinoflagellates, while previous studies have shown the protein expression of single-domain PKSs, this is one of the first pieces of evidence showing the protein production of a multi-modular polyketide synthase [16]. However, since we do not see evidence of the whole protein being produced, we believe there may be some processing or post-translational modifications occurring. We plan to continue to study this protein using mass spectrometry-based proteomics to fully characterize the protein and any modifications that may be occurring, as well as investigate its involvement in amphidinol synthesis. 

## 4. Materials and Methods

### 4.1. Cell Culture Conditions for A. carterae and A. sanguinea 

*Amphidinium carterae* (CCMP 1314) was grown in ESAW medium modified to contain 10 mM of HEPES in polystyrene culture flasks from Corning (Corning, NY, USA) [39]. The cultures were maintained under constant light at 150 μm cm^−2^ s^−1^ with a 14:10 light:dark schedule. *Akashiwo sanguinea* was grown in the same conditions; however, the ESAW medium was used at a salinity of 15 parts per thousand. 

### 4.2. ^14^C Acetate Labeling of A. carterae and A. sanguinea

The cultures of *A. carterae* and *A. sanguinea* were cultured as described above. Cells were counted using a Coulter counter and had cell densities of 79,000 cells/mL for *A. carterae* and 1000 cells/mL for *A. sanguinea*. All experiments were conducted in a light room that had a light intensity of 100 μm cm^−2^ s^−1^. For each experiment, 2 mL of the culture was aliquoted to a clean test tube, and experiments were conducted in triplicate. Treatment groups received cerulenin (Sigma-Aldrich, Saint Louis, MO, USA) at a concentration of 100 μM for *A. carterae* and 45 μM for *A. sanguinea* at 3 time points: 0 h, 1 h, and 2 h. Control groups received 11 μL of DMSO at the same 3 time points: 0 h, 1 h, and 2 h. Samples were incubated with cerulenin and DMSO for 5 min prior to acetic acid [1-^14^C] with a specific activity of 55.2 mCi/mmol (Lot# 184901) (Perkin Elmer, Waltham, MA, USA) addition. A total of 10 μCi of ^14^C acetic acid was added to each sample and was inverted three times until mixed. Initially, T0-hour test tubes were immediately placed in an ice bath to stop the reaction. The T 1 h and T 2 h test tubes were placed in ice baths after 1 and 2 h incubations were complete, respectively. After the cessation of all the reactions, the test tubes were spun down at 800× *g* for 20 min. The supernatant was poured into a clean test tube and stored at 4 °C. The pellet was stored at −20 °C. 

### 4.3. Lipid and Toxin Extraction from Cell Pellets of A. carterae and A. sanguinea

Lipid extraction followed the protocol as described by Adolf et al. 2007 [40]. In total, 1 mL of methylene chloride:methanol at a 2:1 ratio was added to the pelleted samples and vortexed for 10 s. In total, 1 mL of methylene chloride at a ratio of 1:1 and 1 mL of methylene chloride at a ratio of 1:2 were added, and the samples were vortexed for 10 s. Test tubes were placed in a rack until phase separation occurred. Aqueous phases, which contained the toxins, and organic phases, which contained lipids, were separated and placed in clean test tubes for further analysis. 

### 4.4. Sample Preparation for LC/MS Analysis 

The aqueous fraction from the lipid extraction, as described above, was used for toxin analysis. The samples were dried down in a Speed Vac to complete dryness. Samples were then re-suspended in 1 mL of a 30% methanol solution for injection into the LC/MS. 

### 4.5. Thin Layer Chromatography (TLC) Analysis of Lipids in A. carterae and A. sanguinea

In total, 400 μL of the extracted lipids were dried down in a speed vac at a low drying rate until completely dry. The samples were resuspended in 10 μL of methylene chloride:methanol at a 1:1 ratio. Samples were dotted onto a silica HPTLC-GHL plate (Uniplate™, Newark, DE, USA) in 3 μL increments, allowing the samples to dry in between their application. The plate was placed in a tank containing a migration solution of hexane: diethyl ether: formic acid at a ratio of 80:20:2 for 30 min. The TLC plate was dried in an oven at 70 °C until completely dry. The plate was exposed to a phosphor imager screen for 3 days and imaged on a Typhoon Imager (GE Healthcare, Marlborough, MA, USA).

### 4.6. Cell Culture and Harvest for Diel RNA Quantification

*Amphidinium carterae* NCMA, strain number 1314, was grown in an ESAW medium [39], which was modified to contain 10 mM HEPES in a 20 L multiport polycarbonate carboy. The cultures were maintained under constant light at 150 μm cm ^−2^ s^−1^ with a 14:10 light:dark schedule, and the bubbling of air infused with CO_2_ controlled by an American Marine (Ridgefield Ct) pH controller was set to turn on above pH 8.2 and turn off at pH 7.6. Two liters of the medium were added to an actively growing culture on Monday, Wednesday, and Friday until a cell density of 172,000 cells/mL was reached on the morning of the experiment. In total, 250 mL of culture was aliquoted into 26.75 cm^2^ polystyrene culture flasks from Corning (Corning, NY, USA) and placed along the light bank to achieve equivalent light exposure as the stock culture in two rows of thirteen with duplicates in an opposing direction. Each duplicate pair was taken for harvest according to the following schedule relative to the transition from light to dark in hours: −6.0, −4.0, −2.0, −1.5, −1.0, −0.5, 0, 0.5, 1.0, 1.5, 2.0, 4.0, 6.0. The samples were each split into 50 mL and 200 mL aliquots. Each aliquot was centrifuged at 1000× *g* for 10 min at 20 °C. The 200 mL aliquot was frozen at −80 °C, and the 50 mL aliquot was resuspended in 1 mL of the tri reagent (Sigma-Aldrich, Saint Louis, MO, USA).

### 4.7. RNA Extraction and qPCR Analysis

Duplicate samples from each timepoint were extracted using the tri reagent according to the manufacturer’s protocol using 1 mL of the tri reagent. RNA was quantified on a Nanodrop 1000 (Thermo Fisher by Life Technologies Waltham, MA, USA) and also on a Qubit 2.0 fluorometer (Life Technologies). RNA was reverse transcribed using Superscript II reverse transcriptase (Invitrogen by Life Technologies) with Random Primers (Invitrogen) according to the manufacturer’s protocol. Generated cDNA was used as a template for quantitative real-time PCR using an Applied Biosystems (Life Technologies) Fast 7500 thermal cycler in duplicate with the following reaction setup and primers listed in Table 3: 6 μL of diethylpyrocarbonate (DEPC) treated water, 2 μL of combined with forward and reverse primers at 5 μm each, 10 μL of itaq 2X master mix containing sybr green and ROX (Bio-Rad, Hercules, CA, USA), and 2 μL of template cDNA at 10 ng/μL. Thermal cycling conditions consisted of an initial denaturation at 95 °C for 2 min followed by 40 cycles of denaturation at 95 °C for 15 s, annealing and fluorescent data collection at 60 °C for 15 s, and extension at 72 °C for 30 s. The reaction was completed with a melt curve to determine the presence of spurious PCR products. Cycle thresholds and baselines were determined manually, and relative quantities were determined across the diel time course using a Δ-Δ calculation relative to the large ribosomal targeted products and assuming a primer efficiency of 1.85 copies/cycle. This was conducted for normalization across the time course and not to produce accurate quantities; thus, primer efficiencies were not determined empirically for each primer pair. 

### 4.8. Cell Culture and Harvest for SDS-PAGE and Mass Spectrometry

*Amphidinium carterae* was cultured as described above. Cultures were grown to a high cell density (300,000 cells/mL or higher) as measured with a hemocytometer. Cells were collected at midday time points. The aliquots (50 mL) of culture were poured into a 50 mL conical tube and centrifuged at 350× *g* for 10 min at room temperature. The supernatant was poured off, and the tube containing the pellet was placed in a bath containing ethanol and dry ice until frozen. The samples were then stored at −80 °C until analysis. 

### 4.9. Western Blotting

Cell pellets of *A. carterae* were re-suspended in 1 mL of the 1X SDS sample buffer (50 mM Tris-Cl pH 6.8, 2% SDS, 0.1% bromophenol blue, 10% glycerol, 100 mM dithiothreitol), which were incubated for 5 min at 95 °C, and centrifuged at 10,000× *g* for 5 min. The samples were loaded onto gel at varying cell equivalents per lane and separated on Novex™ NuPAGE 3–8% tris-acetate gels at 150 V (constant) for 1 h. Proteins were transferred onto a PVDF membrane using the high molecular weight program on the Trans Blot^®^ Turbo™ Transfer System (Bio-Rad, Hercules, CA, USA) for 14 min. The used transfer buffer was poured out, and a fresh cold transfer buffer was pipetted in without opening up the cassette. The high molecular weight program was run for an additional 7 min. Membranes were washed briefly with TBS-T (50 mM Tris base, 150 mM NaCl, pH 7.4, and 0.05% (*v*/*v*) Tween 20). Membranes were incubated with a primary antibody (Table 2) in TBS-T containing 5% non-fat dry milk overnight at 4 °C with gentle agitation. The membrane was washed six times for 10 min each in TBS-T. Membranes were incubated with the appropriate HRP-conjugated secondary antibody diluted in TBS-T containing 5% non-fat milk for 1 h at room temperature with gentle agitation. The membrane was washed six times for 10 min each in TBS-T. Blots were incubated for 5 min in a Clarity Western ECL Substrate (Bio-Rad, Hercules, CA, USA) according to the manufacturer’s protocol and imaged on the ChemiDoc Touch Imaging System by Bio-Rad. Image processing and molecular weight analysis were conducted using Image Lab™ Software by Bio-Rad.

### 4.10. HPLC and LC–MS Analysis of Amphidinol

LC–MS was performed using an Agilent 1100 Series LC-MSD system comprising a binary pump system, an autosampler, and a diode array detector (DAD) with a micro high-pressure flow cell (6 mm pathlength, 1.7 μL volume), fraction collector, and quadrupole mass spectrometer (G1956A SL) equipped with an electrospray–ionization (ESI) interface. Toxin samples in 30% methanol/water solutions were injected onto a C8 reversed phase (LiChrosphere 125 mm × 4 mm, 5 μm bead size RP-8, Agilent, Santa Clara, CA, USA) column and were subjected to a 1 mL min^−1^ binary methanol/water gradient from 10 to 95% methanol over 25 min. The DAD was operated over the wavelength range of 190–950 nm. Based on the UV–vis spectra, the absorption at 280 nm was used to detect amphidinol. The entire UV–vis spectra were saved for each detectable peak. The eluate from the DAD was split (1/3 to 1/6) using a graduated micro-splitter valve (Upchurch Scientific, Lake Forest, IL, USA). The major portion of the eluate was collected in multiple fractions while the remaining portion was subjected to MS analysis under the following spray chamber conditions: a drying gas (N_2_) flow rate of 10 L min^−1^, pressure 60 psi, the temperature at 350 °C, a fragmentor voltage of 350 V, a capillary voltage of 4000 V. A 1% (*v*/*v*), and formic acid in the water solution (0.1 mL min^−1^) was added post-column via a T-connector to provide lower pH conditions for enhanced positive mode ionization. A 5 mM ammonium acetate solution in the water was used for negative mode ionization. Fractions of 20 s of duration (1/3 mL) during the first 32 min of the HPLC run were collected into scintillation vials to count disintegrations per minute (dpm) in fractions and quantify toxin concentrations. Total ion chromatograms for ions in the range from *m*/*z* 500 to 1500 were collected.

For routine screening, the gradient was shortened (compared to the method described above) by starting with 30% of methanol held isocratically for 3 min; this was then ramped to 60% over 4 min and 90% over 10 min and, finally, held at isocratically at 95% for 5 min with a 3 min return to 30% methanol, thus reducing the runtime from 40 to 26 min. In addition, the column eluent was diverted from the DAD and MS for the first 2 min to help avoid excess salt buildup from the culture media in the spray chamber. These conditions made the initial solvent conditions the same as the sample solvent mixture and improved peak shape.

### 4.11. KS peptide and Cerulenin Adducts

A synthetic peptide was synthesized with the amino acid sequence: DTACSS by Genscript^®^ (Piscataway, NJ, USA). The peptide was dissolved in Optima^®^ LC/MS water (Fisher Chemical, Waltham, MA, USA) to a concentration of 1 mg/mL. Reactions were conducted in 2 mL amber autosampler tubes from Agilent (Agilent, Santa Clara, CA, USA). All reaction components were added to the tube and allowed to incubate at room temperature for 2 h and were then placed at 4 °C until mass spectrometry analysis. Reactions are included in Table 4. 

### 4.12. MS Analysis of Synthetic Peptide with Cerulenin

Peptides were dissolved in Optima^®^ LC/MS water (Fisher Chemical, Waltham, MA, USA) at a concentration of 1 mg/mL. Peptide solutions were infused at a flow rate of 10 µL min^−1^ through the electrospray ionization source of a LCMS IT-TOF mass spectrometer (Shimadzu Scientific Instruments, Columbia, MD, USA). The CDL and heat block were set at 200 °C. Nebulization gas was set to 1.0 L min^−1^, and drying gas was set to “on”. Accurate mass spectra were collected in the positive ionization mode from *m*/*z* 200 to 2000. Tandem mass spectra were acquired using ultra-pure argon (Airgas, Alexandra, VA, USA) as the collision gas at an arbitrary collision energy setting of 40% and an arbitrary flow rate of 50%. The precursor ion accumulation time in the ion trap was set at 10 ms. Raw data files were processed in LCMSSolution software version 3.7 (Shimadzu Scientific Instruments, Columbia, MD, USA) and, when necessary, converted to the mzXML format using the LCMS Solution convert tool. Peak picking and theoretical product ion *m*/*z* generation were performed in mMass v5.5 [41].

## Figures and Tables

**Table 1:** Percent incorporation of ^14^C acetate into total lipids, sulpho-amphidinol, and amphidinol.

Species	Time Point (h)	Cerulenin (+/−)	% In Lipids	% In Sulpho-amphidinol	% In Amphidinol
*A. carterae*	0	+	0.110	0.020	0.0003
*A. carterae*	1	+	0.524	0.038	0.0005
*A. carterae*	2	+	0.058	0.002	0.0008
*A. carterae*	0	−	0.352	0.097	0.0004
*A. carterae*	1	−	0.702	0.014	0.0020
*A. carterae*	2	−	1.351	0.058	0.0035
*A. sanguinea*	0	+	0.002	---	---
*A. sanguinea*	1	+	0.198	---	---
*A. sanguinea*	2	+	0.325	---	---
*A. sanguinea*	0	−	0.230	---	---
*A. sanguinea*	1	−	0.442	---	---
*A. sanguinea*	2	−	0.691	---	---

Table shows the percentage of incorporation for μCi of ^14^C acetate incorporated into lipid and toxin fractions during the labeling experiment. Cerulenin addition is denoted by (+) and control samples are denoted with (−). Numbers are shown as percentages.

**Table 2:** Epitope sequence of antibodies synthesized by GenScript as well as concentrations of primary and secondary antibodies used in Western blotting experiments.

Primary Antibody	Peptide Sequence	Dilution	Secondary Antibody	Dilution
*A. carterae* KS1	CSFPGNAGGAQRY	1:2000	Goat Anti-rabbit IgG-HRP Conjugate (Bio-Rad)	1:2500
*A. carterae* KR2	SRSGKVQPGFGLEGC	1:2000	Goat Anti-rabbit IgG-HRP Conjugate (Bio-Rad)	1:2500
*A. carterae* TE	KEVPVRQVPGGHFGC	1:2000	Goat Anti-rabbit IgG-HRP Conjugate (Bio-Rad)	1:2500

**Table 3:** Primer sequences used in RT-PCR experiments.

Primer Name	Forward Primer Sequence	Reverse Primer Sequence
*A. carterae* LSU	GGCGATGAGGGATGAACCTA	ACCACCGTCCTGCTGTCAGT
*A. carterae* ITS	ATGGCGAATGAAAGGAGATG	AGGGATGACAGATGCCAGAC
*A. carterae* Triple KS (beginning)	GACTTCATTTGGTCGCAGGT	TCAGCCAAGTCGTTTGTGGAG
*A. carterae* Triple KS (end)	ACAGGCCTTGTTGACAGCTT	ACGTCGCACAGCTTTTTCTT

**Table 4:** Experimental set up of synthetic peptide experiments analyzed by mass spectrometry.

Sample Name	Peptide (µg)	50 mM Ammonium Bicarbonate pH 8 (µg)	Adduct Compound (µg)
Peptide Alone	50	50	50
Peptide + Iodoacetamide (IAA)	50	50	50
Peptide + Cerulenin	50	50	50
Peptide + Short Cerulenin	50	50	50

## Data Availability

The GenBank accession number for *Amphidinium carterae* PKS sequence is OR267170 and for *Akashiwo sanguinea* is OR059194. These sequences can be accessed here: https://www.ncbi.nlm.nih.gov/genbank/ (accessed on 30 May 2023).
